# Online and Face-to-Face Social Networks and Dispositional Affectivity. How to Promote Entrepreneurial Intention in Higher Education Environments to Achieve Disruptive Innovations?

**DOI:** 10.3389/fpsyg.2020.588634

**Published:** 2020-12-17

**Authors:** Héctor Pérez-Fernández, Natalia Martín-Cruz, Juan B. Delgado-García, Ana I. Rodríguez-Escudero

**Affiliations:** ^1^Department of Business and Marketing, University of Valladolid, Valladolid, Spain; ^2^Department of Business Economics, University of Burgos, Burgos, Spain

**Keywords:** disruptive innovation, entrepreneurial intention, social networks, dispositional affectivity, digital transformation

## Abstract

Although entrepreneurial intention has been widely studied using cognitive models, we still lack entrepreneurial vocation and, therefore, lack disruptive innovations. Entrepreneurship scholars have some understanding of the reasons underlying this weakness, although there is much room for improvement in our learning concerning how to promote entrepreneurship among university students, especially in the transformed context of digital technologies. This paper focuses on the early stages of start-up, and in particular seeks to evaluate what role social and psychological factors play in the development of entrepreneurial intentions. Drawing on network theory, we consider the impact of social networks on entrepreneurial intention. Specifically, we analyze the influence of two types of social networks: face-to-face and online social networks, with the latter proving especially important in digital transformations. In addition, based on affective congruency theory, we relate affect with entrepreneurial intention. Particularly, we evaluate the influence of positive and negative dispositional affectivity on the formation of entrepreneurial intentions. Finally, since affect and emotions can also be related with social relationships, we analyze whether dispositional affectivities influence entrepreneurial intention through the mediation effect of social networks. Using structural equation modeling, we confirm the impact of both online and face-to-face social networks, as well as positive dispositional affectivity on entrepreneurial intention for 589 higher education students in Spain. However, negative dispositional affectivity is not seen to influence entrepreneurial intention. Furthermore, both face-to-face and online social networks are influenced by positive dispositional affectivity. Moreover, these two types of networks can even partially mediate the relationship between positive dispositional affectivity and entrepreneurial intention. Positive dispositional affectivity can thus influence entrepreneurial intention in two different ways: directly and indirectly through both face-to-face and online social networks. This study provides further insights and adds to the literature on affect, social networks, and entrepreneurial intention. From a broader perspective, we also contribute to the literature on disruptive innovations by explaining how the development of entrepreneurial intentions would have positive consequences for university students vis-à-vis achieving these disruptive innovations.

## Introduction

Disruptive innovation is irremediably linked to entrepreneurship (Schumpeter, 1934), being considered as the underlying driver of the disruptive phenomenon (Chandra and Yang, 2011). In fact, disruptive innovations and entrepreneurship are key factors for the economic and social development (Si et al., 2020). However, entrepreneurial intentions, which are the intentions to start a new company (Krueger et al., 2000), are low, especially in the countries where the income is high. For instance, according to Global Entrepreneurship Monitor (2018), the average percentage of individuals among 18 and 64 years that manifest their intentions to start up is 20.4%. These low entrepreneurial intentions hinder disruptive innovations. This is particularly important in universities, where entrepreneurial intentions are even lower since only the 9.0% of all students intend to be an entrepreneur after finishing their studies (Global University Entrepreneurial Spirit Student’s Survey, 2018). Therefore, there is a need to boost the entrepreneurial intention of university students because they have specialized knowledge and competences (Galloway and Brown, 2002). More specifically, they have knowledge and competences in terms of new technologies and Internet (Venkatesh and Morris, 2000), which are particularly valuable vis-à-vis creating disruptive innovations. Entrepreneurial intentions are crucial to understanding entrepreneurship, involving careful planning and thinking by the individual in a cognitive way (Bird, 1988). Traditionally, entrepreneurial intention has been studied with different cognitive models derived from psychology, such as the entrepreneurial event model (Shapero and Sokol, 1982) or theory of planned behavior (Ajzen, 1991).

However, these cognitive models fail to fully take account of the fact that individuals are influenced by their environment. As argued by Bandura (1986), cognition is not isolated in internal processes of individuals because it is interdependent with their physical and social environment. In this sense, previous research has considered that social environment interacts with individuals to boost the discovery, exploration and exploitation of opportunities (De Carolis and Saparito, 2006; Corbett, 2007). One key element of a person’s social environment is their social networks (face-to-face and online) since individuals maintain social relationships with a large number of other people (Hoang and Antoncic, 2003). Face-to-face networks are based on physical relationships that individuals maintain in their daily lives over long periods (Gedajlovic et al., 2013). Network theory has explained the key role played by face-to-face social networks in entrepreneurship (e.g., Hoang and Antoncic, 2003; Jack, 2010). Nevertheless, the Internet, and particularly social network sites (SNSs) such as Facebook or Twitter, have changed physical relationships, especially for university students since the latter use online social networks extensively to connect with other people (Subrahmanyam et al., 2008). Moreover, these online social networks can promote business innovations through information and knowledge sharing (Pérez-González et al., 2017), thereby supporting entrepreneurial activities (Smith et al., 2017). Since literature suggests that face-to-face and online social networks may be different constructs with different consequences (Gil de Zúñiga et al., 2017), we address the influence of both face-to-face and online social networks in entrepreneurial intention.

Furthermore, over the last decade interest has arisen vis-à-vis the role of affect and emotions in entrepreneurship. Traditional psychological studies consider the association of affect and cognition (Zajonc, 1980; Lazarus, 1982), suggesting that any analysis of an individual’s cognition requires a careful understanding of their emotions (Forgas, 1995). Drawing on this argument, entrepreneurship research has considered the relevance of affect on entrepreneurial processes (Baron, 2008; Delgado-García et al., 2015), such as opportunity evaluation (Foo, 2011), opportunity exploitation (Grichnik et al., 2010) and self-employment transitions (Nikolaev et al., 2019). Despite these studies, the earlier stages of entrepreneurial process have received less attention by previous literature (Delgado-García et al., 2015). Therefore, we address this gap by building on affective congruency theory (Rusting, 1998) in order to explore the role of affect of potential entrepreneurs. This theory explains that individuals process more efficiently the information which is in line with their affect (Rusting, 1998). Among the different concepts associated with affect, we focus on dispositional affectivities defined as stable tendencies to experience positive or negative affect in the long-term (Baron, 2008) because these stable tendencies are relevant for entrepreneurial decisions (Nikolaev et al., 2019). Additionally, given the inherent characteristics of entrepreneurship, an individual’s intention to become an entrepreneur does not develop over a short period, particularly with regard to the uncertainty and personal risk involved in entrepreneurship (Baron, 2008).

Therefore, we hypothesize that dispositional affectivities influence entrepreneurial intention in the same direction as affective valence (positive or negative).

Finally, individual differences in affect can have important consequences in social relationships (Keltner, 1996). Indeed, previous entrepreneurship literature suggests that affect could be one antecedent of individuals’ social networks, which may have different consequences on entrepreneurship (Baron, 2008; Hayton and Cholakova, 2012). Therefore, we address this by conjecturing that dispositional affectivities may influence entrepreneurial intention, not only because of the role they pay in individuals’ cognition, but also by influencing the development of their social networks. Our research explores whether affect and emotions provide the first step toward developing face-to-face and online social networks which, in turn, might influence entrepreneurial intentions; i.e., the relationship between affect and entrepreneurial intention and whether this relation is mediated by social networks. We test these hypotheses in a sample of 589 university students from two universities in Spain.

This research makes various contributions to entrepreneurship research. First, we advance research by considering how social networks influence entrepreneurial intention. In particular, we analyze what influence social network size (both online and face-to-face) has on entrepreneurial intentions. Therefore, we simultaneously consider both the social environment and entrepreneurial cognition, and provide a fuller explanation than those which simply examine either one or the other (De Carolis and Saparito, 2006; De Carolis et al., 2009). Second, we extend prior research on the role of affect in entrepreneurship by considering the influence of dispositional affectivities on entrepreneurial intention, beyond traditional cognitive intention models (Shapero and Sokol, 1982; Ajzen, 1991). In this sense, our study follows the recommendation of Baron (2008) with regard to exploring the interaction of affect and cognition in an effort to enhance research on entrepreneurial cognition. Third, we also contribute to the research on entrepreneurial intention by analyzing the dual role of dispositional affectivities in entrepreneurial cognition and by revealing the cognitive and social mechanisms that underlie this influence. We thus respond to Fayolle and Liñán’s (2014) suggestion to expand the antecedents, moderators, and mediators of entrepreneurial intention in order to increase our knowledge thereof. Finally, from a broader perspective, we contribute to the literature on disruptive innovations, which has found that SNSs promote disruptive innovations in established companies (Scuotto et al., 2017). We extend this to previous stages of the start-up process by explaining how SNSs, in conjunction with dispositional affectivities, encourage disruptive innovations through the development of individuals’ entrepreneurial intention. In this sense, entrepreneurship theories may be a unique source of insights for advancing in the study of disruptive innovations since the objective of study would be evaluated differently (Christensen et al., 2016).

## Theoretical Background

Social networks consist on “a set of actors and some set of relationships that link them” (Hoang and Antoncic, 2003). These social relations are a fundamental element of everyone’s life (Kim and Aldrich, 2005). Individuals currently have two types of social networks: face-to-face and online. Face-to-face are the physical networks that people have in their daily lives over long periods (Gedajlovic et al., 2013). Nevertheless, SNSs are key to supplementing these physical networks. SNSs such as Facebook or Twitter are web-based services where individuals construct a public profile within a system, articulate a list of other individuals that share a connection with them and view their list of connections and the lists of others (Boyd and Ellison, 2007). Therefore, SNSs create a context that favors meaningful communicative exchanges and potential benefits (Ellison et al., 2014).

Previous research on entrepreneurship has found that face-to-face social networks influence the different entrepreneurial processes and outcomes (Hoang and Antoncic, 2003; Jack, 2010). These studies have usually employed network theory arguments that social networks have a significant impact on the type and extent of resources acquired by entrepreneurs (Jack, 2005). Therefore, these social networks may contribute earlier, developing a willingness to create a new company, which has not been widely studied in entrepreneurship (Bonesso et al., 2018). Furthermore, online social networks have scarcely been considered in entrepreneurship research (Smith et al., 2017), even though entrepreneurs increasingly use these SNSs (Sigfusson and Chetty, 2013; Fischer and Reuber, 2014). SNSs offer an unprecedented opportunity for entrepreneurs to participate in interactions on a scale and in a manner not previously possible and to access new information (Reuber and Fischer, 2011). In this sense, SNSs provide an efficient and effective means to grow a business (Edosomwan et al., 2011). For instance, entrepreneurs obtain knowledge in SNSs that helps to foster innovations in small and medium-sized enterprises (Candi et al., 2018; Papa et al., 2018). Affect refers to the general phenomenon of subjective feelings (Barsade, 2002). The general phenomenon of subjective feelings includes different types of experiences such as dispositional affectivity, specific emotion, and mood. As previously commented, dispositional affectivities are stable tendencies to experience positive or negative affect in the long-term (Baron, 2008). Specific emotions are the consequence of specific events. They disappear quickly and are characterized by being highly intense. Conversely, moods are not associated to specific events, are stable and involve low intensity (Frijda, 1986). Both specific emotions and moods are affective states. Traditional research on affect has considered the impact of affect on cognition by examining the impact of affective valence (positive or negative) (Forgas, 1995; Rusting, 1998). In particular, previous research on affect has explored affective congruence arguments, which predicts that individuals process more efficiently the information that is in line with their affects. In other words, when an individual has positive or negative affect, it will be easier for him or her to perceive, attend to, learn and interpret information of the same emotional valence (Rusting, 1998). Regarding this theory, dispositional affectivities and affective states produce similar effects across situations (Rusting, 1998; Lyubomirsky et al., 2005).

Previous research on entrepreneurship has evidenced the important impact of affect and emotions on entrepreneurial cognition and decision-making (Baron, 2008; Delgado-García et al., 2015). Baron (2008) was the first to propose the role of affect in different key aspects of entrepreneurship. After this work, some authors have explored the influence of affect on different entrepreneurial processes. These authors have mainly focused in the more advanced steps of entrepreneurship (Delgado-García et al., 2015). For instance, Foo (2011) have found how emotions influence opportunity risk perception and preferences in opportunity evaluation. In addition, Grichnik et al. (2010) have found that both positive and negative affect condition the allocation of time and resources to exploit an entrepreneurial opportunity. Finally, Nikolaev et al. (2019) have examined how positive and negative dispositional affectivities influence entry into entrepreneurship.

## Hypotheses Development

### Dispositional Affectivities and Entrepreneurial Intention

Based on affective congruency (Rusting, 1998), positive affect can influence the interpretation of situations more positively (Isen et al., 1978; Isen and Shalker, 1982), leading individuals to overestimate the chances of positive outcomes (Wright and Bower, 1992; Zelenski and Larsen, 2002). In the entrepreneurial context, positive affect would encourage individuals to expect better outcomes if they decide to start up (Simon et al., 2000). In addition, when evaluating the possible outcomes of entrepreneurship, individuals take into account the inherent risks involved in entrepreneurship. Positive affect decreases how individuals consider the possibility of risks (Wright and Bower, 1992), such that they would see entrepreneurship as being less risky than it really is because they would consider the positive information about entrepreneurship from their memory (Isen et al., 1985). Finally, positive affect makes individuals trust on their knowledge (Bless et al., 1996; Foo et al., 2015) and skills (Baron, 2007), including their entrepreneurship-related knowledge and skills. Therefore, we propose:

H1: Individuals’ positive dispositional affectivity is related to greater entrepreneurial intention.

Based on affective congruency (Rusting, 1998), negative affect can promote negativity bias, which is a propensity to overestimate the relevance of negative information regarding any situation (Kunda, 1999), thus triggering pessimistic evaluations (Direnfeld and Roberts, 2006). Hence, negative affect leads individuals to overestimate the possibility of negative results (Wright and Bower, 1992; Zelenski and Larsen, 2002), including the possible negative outcomes of entrepreneurship. Furthermore, negative affect leads individuals to perceive situations as threatening, such that they seek to avoid potential losses (Jorgensen, 1996). Thus, negative affect can lead individuals to consider entrepreneurship as a future high-risk option because this affect lead to the activation of negative associations and memories, influencing the judgment of the risk of entrepreneurship (Baron, 2008). Finally, negative affect influences individuals’ consideration of their capabilities in a deficient manner, leading them to evaluate their knowledge (Ambady and Gray, 2002). Individuals’ negative affect is therefore associated with a reduced sense of control regarding task management (Bosma et al., 1998), including the tasks required to engage in entrepreneurial behavior. Taking into account these arguments, we propose that:

H2: Individuals’ negative dispositional affectivity is related to less entrepreneurial intention.

### Dispositional Affectivities and Social Networks

Previous literature has considered that positive affect promotes the appearance of social relationships (Lyubomirsky et al., 2005). First, positive perspectives in individuals allow them to be more attractive in an interpersonal way (Staw et al., 1994) such that other individuals want to be with them (Srivastava et al., 2006). In fact, individuals seek communication with others who display positive affect (Watson et al., 1992; Berry and Hansen, 1996) since they believe that these social interactions will allow them to obtain greater rewards (Harker and Keltner, 2001).

Additionally, positive affect increases individuals’ tendency to seek new and more varied social ties (Lucas and Diener, 2003; Andersson, 2012). In this sense, previous research has associated positive affect (Baron, 2008) and happiness (Requena, 1995) with differences in individuals’ social networks, for example in the size of these social networks. Therefore, we propose:

H3a: Individuals’ positive dispositional affectivity is related to them having larger face-to-face social networks.
H3b: Individuals’ positive dispositional affectivity is related to them having larger online social networks.

Previous research has found that negative affect decreases social abilities (Mor and Winquist, 2002). Additionally, negative affect reduces how many social interactions an entrepreneur has (Baron, 2008) since other individuals prefer to interact less in social relationships with high negative affect individuals (Staw et al., 1994). This is because individuals’ social relationships that involve negative affect typically tend to be unpleasant (Berry and Hansen, 1996). Furthermore, these individuals are less likely than others to initiate a conversation (Cunningham, 1988). They therefore interact less in social terms and, when they do socially interact, these interactions are more negative (Räikkönen et al., 1999) and shorter (Geers et al., 1998). Following this, previous literature has suggested that higher negative affect individuals tend to have smaller social networks (Lucas and Diener, 2003). Hence, we propose:

H4a: Individuals’ negative dispositional affectivity is related to them having smaller face-to-face social networks.
H4b: Individuals’ negative dispositional affectivity is related to them having smaller online social networks.

### The Mediating Role of Social Networks

Previous research considers that the effects of personal dispositions are often related to their interaction with environmental factors (Wood and Bandura, 1989). In this sense, previous literature on entrepreneurship considers that the relationships between affect (for example, dispositional affectivities) and cognitive processes (for example, entrepreneurial intention) occur in a context of moderating and mediating environmental variables (Hmieleski and Baron, 2009). In addition, Baron (2008) proposes that affect can influence the frequency or quality of social contacts, which may have consequences on entrepreneurship through the access to essential resources for entrepreneurs that these social networks provide. Hayton and Cholakova (2012) develop propositions regarding the influence of human capital, time invested, idea complexity or relevance to core self in the relationship between affect and the intention to develop an entrepreneurial idea. Beyond these propositions, they also suggest that affect may not only influence entrepreneurship directly through individual cognitive processes, but also more indirectly due to its influence in terms of developing the social networks through which individuals can obtain relevant information and resources. Therefore, the impact of dispositional affectivities on entrepreneurial intention may not only be the result of an individual cognitive process, but also a consequence of the mediation effect of social networks.

Drawing on the network theory, previous research has shown how prior contacts, especially friends or family, may provide resources in the start-up (Johannisson, 1988), which proves relevant in the early stages of creating a new business (Greve and Salaff, 2003). The most intuitive network component is size, i.e., the number of links between a central individual and others (Hoang and Antoncic, 2003). Entrepreneurs try to extend social networks so as to acquire important information and resources (Greve and Salaff, 2003). In fact, individuals with a larger network are well positioned to acquire the resources required for their entrepreneurial activities (Dubini and Aldrich, 1991), allowing them to have greater control over entrepreneurship (De Carolis et al., 2009). Finally, De Carolis and Saparito (2006) find that networks consisting of many contacts reduce uncertainty in exchanges, which increases an individual’s belief that they will achieve the expected outcomes, making the pursuit of a new entrepreneurial opportunity more attractive. We thus propose:

H5a: The greater the size of the face-to-face social networks, the greater the entrepreneurial intention.

SNSs allow individuals to create larger and more disperse social networks (Wellman et al., 2001; Donath and Boyd, 2004) since they can interact with more individuals than they were formerly able to (Ellison et al., 2011). Indeed, as SNSs admit a broader range of individuals, each individual’s networks become larger (Ellison et al., 2014), thereby providing access to different perspectives (Ellison et al., 2014). SNSs offer an infinite number of opportunities to bridge structural holes (Rainie and Wellman, 2012), which in turn increases the possibility of valuable exchanges because these structural holes provide more diverse information (Burt, 2000). Therefore, individuals with more contacts in SNSs view their chances of success in entrepreneurial opportunities positively (Fischer and Reuber, 2011). Therefore, we propose:

H5b: The greater the size of online social networks, the greater the entrepreneurial intention.

We have just explained the direct effect of social network size on entrepreneurial intention, which, together with the explained influence of dispositional affectivities on social networks and the previously mentioned arguments of Baron (2008) and Hayton and Cholakova (2012), allows us to consider the mediating role of these social networks:

H6a: The size of face-to-face networks mediates the relationship between positive dispositional affectivity and entrepreneurial intention.
H6b: The size of online networks mediates the relationship between positive dispositional affectivity and entrepreneurial intention.
H6c: The size of face-to-face networks mediates the relationship between negative dispositional affectivity and entrepreneurial intention.
H6d: The size of online networks mediates the relationship between negative dispositional affectivity and entrepreneurial intention.

**FIGURE 1:**
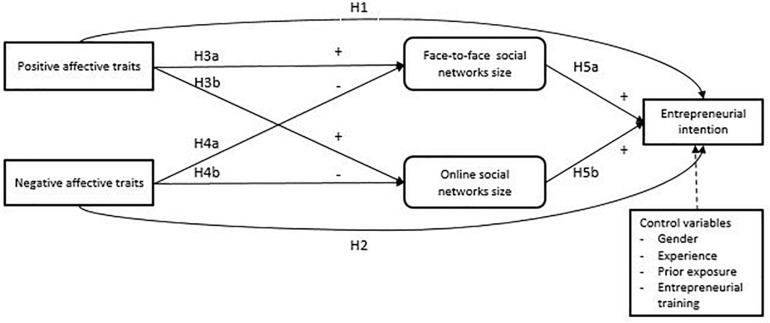
Model of entrepreneurial intention based on social networks and dispositional affectivities.

The model of this study appears in Figure 1.

## Research Method

### Sample and Data Collection

We collected information from October to December 2017 from two public universities in Spain through a cross-sectional design^1^. We obtained 608 responses from students in their final 2 years of university, who answered questionnaires voluntarily after being informed about the objective of the study. The students were taking degrees in business or related disciplines such as finance, accounting, marketing, trade, or economics. Table 1 shows the main characteristics of the final 589 university students in terms of gender, age, experience as self-employed and as an employee, family entrepreneur, and close friend entrepreneur, since 19 responses were removed due to missing data.

**TABLE 1:** Sample characteristics.

Gender	*N*	% of total	Age	*N*	% of total
Male	245	41.6	19	9	1.5
Female	344	58.4	20	90	15.2
			21	200	34.1
			22	125	21.3
			23	69	11.8
			24	28	4.8
			25	25	4.2
			>25	43	7.1
TOTAL	589	100.00	TOTAL	589	100.00

**Experience as self-employed**	***N***	**% of total**	**Experience as employee**		**% of total**

Yes	29	4.9	Yes	300	50.9
No	560	95.1	No	289	49.1
TOTAL	589	100.00	TOTAL	589	100.00

**Family member entrepreneur**	***N***	**% of total**	**Close friend entrepreneur**	***N***	**% of total**

Yes	349	59.3	Yes	293	49.7
No	240	40.7	No	296	50.3
TOTAL	589	100.00	TOTAL	589	100.00

Students in our sample have, on average, roughly 1 year to make a decision regarding their professional career (Fitzsimmons and Douglas, 2011). In this sense, we follow Krueger (1993), who indicates that in order to analyze entrepreneurial intention, researchers must use samples of individuals who are now facing important career decisions. Given this relatively short period of time, students’ entrepreneurial intention is likely to be the same after graduation (Audet, 2004). Additionally, this segment of the population has specific knowledge and competences that could be exploited through new ventures (Galloway and Brown, 2002), favoring disruptive innovations (Chandra and Yang, 2011). For these reasons, student samples are appropriate in studies on nascent entry into entrepreneurship (Hsu et al., 2017), and are highly prevalent in entrepreneurial intention research (Kolvereid, 1996; Krueger et al., 2000; Fitzsimmons and Douglas, 2011).

### Measurement Scales

We measure entrepreneurial intention with five Entrepreneurial Intent Questionnaire items (Liñán and Chen, 2009), based on prior research (Kolvereid, 1996; Krueger et al., 2000). This questionnaire has been widely used in the literature (e.g., Liñán et al., 2011; Ilouga et al., 2014; Karimi et al., 2016).

Online and face-to-face network size are measured through two items: the total number of friends that individuals connect with online or in face-to-face social networks and how many of these friends are contacted frequently, since both aspects are important in terms of these networks (Ellison et al., 2011).

Finally, we measure individuals’ dispositional affectivities with the PANAS scale (Watson et al., 1988), which is a widely used scale in research on affect. We follow the adaptation in Spanish of Sandín et al. (1999). Twenty items make up this scale, with ten items being related to positive dispositional affectivity and ten to negative dispositional affectivity.

### Control Variables and Common Method Bias

We use different control variables to analyze entrepreneurial intention. First, we include age since the literature has shown that age is negatively associated with the propensity for entrepreneurship (Curran and Blackburn, 2001). Additionally, previous findings have reported that women display less inclination toward entrepreneurial activities than men (Mathews and Moser, 1995). We therefore incorporate a gender dummy (1 = female; 0 = male). Furthermore, previous literature has found that both job experience (Mathews and Moser, 1995) and previous experience in entrepreneurship (Shepherd, 2003) are positively associated to the likelihood of starting up. We thus create two different dummy variables (1 = respondent has previous experience as an employee or self-employee, 0 = otherwise). Finally, previous research has found the relationship between entrepreneurial training and entrepreneurial intention (Bae et al., 2014). We therefore incorporate a dummy variable regarding if the university student has received previous entrepreneurial training (1 = yes; 0 = no).

Common method bias can be a severe issue when the dependent and independent variables are measured through the perception and response of the same individual (Podsakoff et al., 2003). In order to assess the severity of this bias, we conducted a Harman one-factor test (Podsakoff and Organ, 1986) with our four main variables in order to ascertain whether variance is largely attributed to any single factor. We adopt the criterion of an eigenvalue greater than 1, and find four factors. The highest covariance explained by one factor is 19.2%. We therefore confirm that said bias is not a concern.

## Analysis and Results

Prior to evaluating the psychometric properties of our scales, we identify the categories of affect using principal component analysis with the varimax rotation (IBM SPSS Statistics 24). Using eigenvalue criteria, we find six categories that are higher or very close to 1: three categories of positive dispositional affectivity and another three of negative dispositional affectivity. These six categories are able to explain the 59.075% of the total variance of positive and negative affect. Table 2 shows the different components of affect that form these six categories as well as the eigenvalues and the percentage of explained variance of each of these categories. This finding is not surprising since there are differences among affects of the same valence (Lerner and Keltner, 2000). Indeed, Watson and Clark (1999) have elaborated the PANAS-X in order to justify that affect is made up of two broad dimensions (positive and negative affect), each of which may consist of various correlated, but ultimately distinguishable specific affects.

**TABLE 2:** Results of PANAS factorial analysis.

PA1	PA2	PA3	NA1	NA2	NA3
λ = 4.112	λ = 0.989	λ = 0.949	λ = 3.312	λ = 1.355	λ = 1.104
% EV = 20.560	% EV = 4.946	% EV = 4.712	% EV = 16.561	% EV = 6.777	% EV = 5.519
Active	Alert	Interested	Afraid	Hostile	Distressed
Enthusiastic	Attentive		Ashamed	Irritable	Jittery
Excited	Determined		Guilty	Upset	Nervous
Proud	Inspired		Scared		
Strong					

*λ, eigenfactor; EV, explained variance of each factor.*

Therefore, individuals can exhibit distinctions on the affect scales regarding a one-dimensional approach for positive and negative affect (Gaudreau et al., 2006). Following this, we consider positive dispositional and negative dispositional affectivities to be second-order constructs, decreasing the number of relationships in this complex structural model, thus making the estimation more parsimonious and easier to understand (Hair et al., 2016).

We employ structural equation modeling for statistical analysis. In particular, we use partial least squares (PLS). This is because PLS has no indeterminacy problems associated with other techniques, does not require data normality (Wittmann et al., 2009), and deal with both first-order and second-order constructs.

### Measurement Model

Since PLS can handle both reflective and formative constructs (Chin and Newsted, 1999), we evaluate the measurement quality of these two different types of constructs. All the first-order constructs are reflective. The second-order constructs of positive and negative dispositional affectivities are formative. Table 3 shows how we measured the first-order constructs. In this Table 3, we also assess the reflective constructs by examining item reliability, internal consistency, as well as convergent and discriminant validity (Roldán and Leal, 2003). Firstly, all items loadings of the first-order constructs are significant at *p* < 0.01 Additionally, all constructs exceed the thresholds for a Cronbach’s alpha of 0.6 and a composite reliability of 0.7. Finally, the average variance extracted also exceeds the threshold of 0.5 (Fornell and Larcker, 1981).

**TABLE 3:** Reliability and convergent validity.

Construct/indicator	Factor loading
**Entrepreneurial intention (α = 0.941, AVE = 0.833, CR = 0.961) Rate the following statements:**	
I am ready to do whatever it takes to become an entrepreneur	0.849**
My professional goal is to become an entrepreneur	0.923**
I will make every effort to create and run my own company	0.924**
I am determined to set up a firm in the future	0.932**
I have seriously thought about starting a business in the future	0.870**
**Online network size (α = 0.607, AVE = 0.716, CR = 0.835)**	
With how many different people, approximately, are you connected through SNSs?	0.855**
With how many of these people do you maintain frequent contact through SNSs?	0.829**
**Face-to-face network size (α = 0.899, AVE = 0.668, CR = 0.923)**	
With how many different people, approximately, are you connected in a personal way?	0.910**
With how many of these people do you maintain frequent contact in a personal way?	0.952**
**Positive affect 1 (α = 0.762, AVE = 0.520, CR = 0.844)**	
Active	0.733**
Enthusiastic	0.694**
Excited	0.729**
Proud	0.658**
Strong	0.763**
Positive affect 2 (α = 0.627, AVE = 0.510, CR = 0.786)	0.678**
Alert	0.649**
Attentive	0.775**
Determined	0.635**
Inspired Positive affect 3 (n.a.) Interested	1.000**
**Negative affect 1 (α = 0.685, AVE = 0.524, CR = 0.814)**	
Afraid	0.682**
Ashamed	0.704**
Guilty	0.710**
Scared	0.763**
Negative affect 2 (α = 0.694, AVE = 0.633, CR = 0.837)	0.807**
Hostile	0.824**
Irritable	0.726**
Upset	0.737**
Negative affect 3 (α = 0.696, AVE = 0.632, CR = 0.837)	0.801**
Distressed Jittery Nervous	0.825**

**p < 0.01.

Beyond the reflective constructs, we evaluate whether each factor contributes significantly to the second-order construct in order to statistically validate their formative character. Table 4 shows the six factors of dispositional affectivities. The outer weights confirm that all the factors are important for the second-order construct. We also verify multicollinearity through the variance inflation factor. There are no collinearity concerns because the values of the factors are below the cut-off value of 5 (Kleinbaum et al., 2013).

**TABLE 4:** Quality criteria of second-order measurement.

Formative second-order construct facets/components	Outer weights	VIF
**Positive affect**		
PA1: excited, strong, enthusiastic, proud, active	0.662**	1.545
PA2: alert, inspired determined, attentive	0.424**	1.557
PA3: interested	0.091**	1.061
**Negative affect**		
NA1: guilty, scared, ashamed, afraid	0.444**	1.337
NA2: hostile, irritable, upset	0.391**	1.393
NA3: distressed, nervous, jittery	0.419**	1.512

Bias-corrected bootstrap significance levels: ** *p* < 0.01 (one-tailed test).VIF, variance inflation factor.

Finally, in Table 5 we evaluate the discriminant validity of the reflective measures by evaluating whether the root square of the average variance extracted is larger than the interconstruct correlations. We support this discriminant validity of our constructs. Summing up, we can affirm that all the constructs display good psychometric properties.

**TABLE 5:** Zero-order correlations and discriminant validity.

	1	2	3	4	5	6	7	8	9	10	11	12	13	14	15
1. Entrepreneurial intention	0.913														
2. Online network size	0.204	**0.846**													
3. Face-to-face network size	0.178	0.402	**0.938**												
4. Positive affect 1	0.259	0.224	0.173	**0.721**											
5. Positive affect 2	0.270	0.131	0.158	0.589	**0.693**										
6. Positive affect 3	0.211	0.069	0.096	0.221	0.204	—									
7. Negative affect 1	−0.002	−0.074	−0.053	−0.087	−0.093	−0.033	**0.723**								
8. Negative affect 2	−0.082	−0.043	−0.018	−0.153	−0.060	−0.035	0.391	**0.795**							
9. Negative affect 3	−0.066	−0.005	−0.086	−0.049	−0.011	−0.081	0.468	0.501	**0.795**						
10. Gender	−0.168	−0.021	−0.193	−0.036	−0.062	0.079	−0.007	0.011	0.072	—					
11. Experience as self-employed	0.135	0.092	0.061	0.136	0.144	0.051	−0.022	−0.013	−0.011	−0.094	—				
12. Experience as employee	0.195	0.091	−0.003	0.140	0.097	0.086	−0.013	−0.039	−0.004	−0.056	0.209	—			
13. Entrepreneur in family	0.150	0.029	−0.009	0.039	0.056	0.080	0.069	0.000	0.037	0.018	0.063	0.078	—		
14. Close friend entrepreneur	0.086	0.161	0.111	0.121	0.142	−0.020	−0.087	−0.040	−0.091	−0.084	0.086	0.166	0.060	—	
15. Entrepreneurial training	0.178	0.119	0.084	0.036	0.097	0.077	0.017	0.002	0.034	−0.109	0.117	0.103	0.005	0.116	—

*The diagonal corresponds to the root square of average variance extracted.*

### Structural Model

We use bootstrapping (2000) in SmartPLS 3.0 to randomly generate subsamples that determine whether the Beta coefficients (β) are significant. Results are shown in Table 6.

**TABLE 6:** Standardized parameter estimates.

Hypotheses	Direct effect	Outcome	
**Direct effects**			
Positive dispositional affectivity → Entrepreneurial intention	0.234***	H1 supported	
Negative dispositional affectivity → Entrepreneurial intention	−0.030	H2 not supported	
Positive dispositional affectivity → Face-to-face social network size	0.188***	H3a supported	
Positive dispositional affectivity → Online social network size	0.210***	H3b supported	
Negative dispositional affectivity → Face-to-face social network size	−0.046	H4a not supported	
Negative dispositional affectivity → Online social network size	−0.030	H4b not supported	
Face-to-face social network size → Entrepreneurial intention	0.061**	H5a supported	
Online social network size → Entrepreneurial intention	0.098***	H5b supported	

**Mediating effects**	**Indirect effect**	**Total effect**	**Outcome**

Positive dispositional affectivity → Face-to-face social network size → Entrepreneurial intention	0.012**	0.266***	H6a partially supported
Positive dispositional affectivity → Online social network size→ Entrepreneurial intention	0.021*	0.266***	H6b partially supported
Negative dispositional affectivity → Face-to-face social network size → Entrepreneurial intention	−0.003	−0.036	H6c not supported
Negative dispositional affectivity → Online social network size → Entrepreneurial intention	−0.003	−0.036	H6d not supported
**Control relationships**			
Gender → Entrepreneurial intention	−0.120***		
Experience as employee → Entrepreneurial intention	0.121***		
Experience as self-employed → Entrepreneurial intention	0.029		
Family member entrepreneur → Entrepreneurial intention	0.126***		
Close friend entrepreneur → Entrepreneurial intention	−0.026		
Entrepreneurial training → Entrepreneurial intention	0.118***		
***R*^2^ of entrepreneurial intention**	**0.190**		
***R*^2^ of face-to-face social network size**	**0.039**		
***R*^2^ of online social network size**	**0.046**		

*** *p* < 0.01, ** *p* < 0.05, * *p* < 0.1. The bold values are referred to the R square in order to show the importance of it.

First, positive dispositional affectivity positively and significantly influences entrepreneurial intention (β = 0.234, *p* = 0.000), thereby supporting H1. However, we do not find support for H2 because negative dispositional affectivity has no significant influence on entrepreneurial intention (β = −0.030, *p* = 0.200). Furthermore, positive dispositional affectivity positively influences both face-to-face social network size (β = 0.188, *p* = 0.000) and online social network size (β = 0.210, *p* = 0.000), such that we find support for H3a and H3b respectively. However, we do not obtain support for H4a and H4b because negative dispositional affectivity has no significant impact on either face-to-face social network size (β = −0.046, *p* = 0.112) or online social network size (β = −0.030, *p* = 0.225). As regards the latter direct effects, entrepreneurial intention is also positively and significantly influenced by both face-to-face social network size (β = 0.061, *p* = 0.048) and online social network size (β = 0.098, *p* = 0.009). Therefore, we find support for H5a and H5b, respectively.

Regarding control variables, results show that men have significantly higher entrepreneurial intention than women (β = −0.149, *p* < 0.001). In addition, previous experience as employee (β = 0.144, *p* < 0.001), having an entrepreneur in their family (β = 0.131, *p* < 0.001) and having previous entrepreneurial training (β = 0.118, *p* < 0.001) are also significant for entrepreneurial intention. However, to have experience as self-employed (β = 0.037, *p* > 0.05) or a close friend entrepreneur (β = 0.030, *p* > 0.05) are not significantly related to entrepreneurial intention. In sum, the control variables have significant effects on entrepreneurial intention.

Finally, to clarify the implications of the previous findings, we obtain in PLS the β of each specific indirect effect of dispositional affectivities on entrepreneurial intention through the size of face-to-face and face-to-face social networks. Thus, we also calculate the total effect of dispositional affectivities on entrepreneurial intention. As Table 5 shows, we obtain four specific indirect effects. First, face-to-face social network size significantly and positively mediates (β = 0.012, *p* = 0.089) the relationship between positive dispositional affectivities and entrepreneurial intention, thus supporting H6a. We also obtain support for H6b because this same relationship is mediated significantly and positively by online social network size (β = 0.021, *p* = 0.028). These mediating effects are partial because, as mentioned, the direct effect of positive dispositional affectivity on entrepreneurial intention is also significantly positive. Adding the two indirect effects and the direct effect, we obtain the total effect of positive dispositional affectivity on entrepreneurial intention (0.266). In contrast, in the relationship between negative dispositional affectivity and entrepreneurial intention, we find no significant mediating role of either face-to-face social network size (β = −0.003, *p* = 0.291) or online social network size (β = −0.003, *p* = 0.345), such that H6c and H6d are not supported. The total effect of negative dispositional affectivity on entrepreneurial intention is −0.036.

## Discussion

This study expands previous research by evaluating the combined influence of affect and social networks on entrepreneurial intention for students in higher education institutions in order to promote new ventures and disruptive innovations among them. First, prior research has found that social networks are a key element when establishing a new firm (Greve and Salaff, 2003; Jack, 2010). Based on networks theory, our findings suggest that both face-to-face and online social networks are also important in the early cognitive steps of entrepreneurship. In this sense, this study responds to De Carolis et al.’s (2009) suggestion that entrepreneurship research can examine how the environment impacts cognition and ultimately affects entrepreneurship.

Second, drawing on affective congruency theory (Rusting, 1998), this research contributes to the research on affect and entrepreneurship by evaluating the importance of dispositional affectivities on entrepreneurial intention. Previous studies have explored the role of affect on several entrepreneurial processes (Baron, 2008; Grichnik et al., 2010; Foo, 2011; Nikolaev et al., 2019), although current understanding of how affect and emotion might impact entrepreneurial cognition, particularly in the early stages of entrepreneurship, remains in its infancy (Delgado-García et al., 2015). We therefore expand previous research into the influence of affect on the first cognitive steps of entrepreneurship. Additionally, we confirm that individuals’ positive affect usually relates to having more extensive social networks than individuals’ negative affect (Lucas and Diener, 2003; Baron, 2008). Thus, these face-to-face and online social networks are a partial mediator of the influence of dispositional affectivities on entrepreneurship, which is line with previous suggestions of literature (Baron, 2008; Hayton and Cholakova, 2012).

From a broader perspective, we contribute to the literature on disruptive innovations by explaining how social networks and dispositional affectivities promote entrepreneurial intentions. Over the past years, disruptive innovation has been widely linked to the study of entrepreneurship (Si et al., 2020). Taking into account that entrepreneurial activity is associated with disruptive innovation (Schumpeter, 1934), the development of entrepreneurial intentions would have positive consequences for university students vis-à-vis achieving these disruptive innovations. In particular, in the current context of digital transformation, individuals can use online social networks to promote disruptive innovations, not only in established companies (Scuotto et al., 2017), but also when creating new companies, as a way of taking advantage of these innovations (Si et al., 2020). In this research, we follow the suggestion of Christensen et al. (2016) regarding the use of entrepreneurship literature in order to advance in the study of disruptive innovations from a different point of view. First, our results show that positive dispositional affectivity positively influences entrepreneurial intention. These results are consistent with Hayton and Cholakova’s (2012) proposition concerning the influence of positive affect on the intention to develop an entrepreneurial idea. In a more general view, these findings are in line with previous literature regarding the importance of positive affect as an element for the cognitive processes of entrepreneurship (Baron, 2008). However, negative dispositional affectivity is seen to have no influence on entrepreneurial intention. Although this finding might at first seem surprising, this is not fully the case. Positive and negative valence of affect do not always produce opposite effects (Lerner and Keltner, 2000). In a recent meta-analysis, Fodor and Pintea (2017) have found a significant positive relation between positive affect and entrepreneurial performance, but the influence of negative affect on entrepreneurial performance is no significant. Our finding could be explained because negative affect encourages individuals to make a greater effort and to engage in a deeper search to identify opportunities (Foo et al., 2015). Individuals’ negative dispositional affectivity would not influence entrepreneurial intention, but might impact subsequent steps of entrepreneurship, given that those who display high negative dispositional affectivity would exhibit entrepreneurial intention (or not), yet might be more cautious than individuals who evidence positive dispositional affectivity.

Furthermore, our results show that both online and face-to-face social networks are positively influenced by positive dispositional affectivity. These results are consistent with previous literature, which has related face-to-face social networks with positive affect (Baron, 2008) and happiness (Requena, 1995). In the case of online networks, we confirm previous research linking psychological well-being, which is related to positive affect, with online network size (Steinfield et al., 2008). Therefore, the ability to be positive within social networks forms a key part of them (Leyden et al., 2014). However, negative dispositional affectivity was found to have no impact on either face-to-face or online networks. As for entrepreneurial intention, positive and negative valence of affect do not always produce opposite effects (Lerner and Keltner, 2000). Indeed, some previous studies have failed to find any relationship between negative affect and social activity (Watson et al., 1992) or have even found a positive relationship between negative affect and social interaction because individuals with negative affect can try to engage in social interactions in order to regulate their negative affect (Berry and Hansen, 1996). Although these studies are based on face-to-face networks, in online networks this situation should be even more pronounced. In these networks, repeated exchanges are more likely because time is compressed, interactions are accelerated, and individuals are more accessible (Baym, 2010). Therefore, individuals with negative affect can interact continuously in order to address their negative affect.

Finally, we discuss the mediating effects of social network size on the relationship between dispositional affectivities and entrepreneurial intention. As regards direct effects, face-to-face social networks positively influence entrepreneurial intention. This result is in line with previous literature concerning the importance of social networks for obtaining resources in the early stages of entrepreneurship (Greve and Salaff, 2003; De Carolis et al., 2009). Our results also show the positive significance of online social networks on entrepreneurial intention. So far, most of the literature on social networks in entrepreneurship has focused on the face-to-face context (Jack, 2010; Gedajlovic et al., 2013). However, the way in which social networks are developed has changed in recent years and the use of online social networks by entrepreneurs forms an important part of their networking activities (Fischer and Reuber, 2011; Sigfusson and Chetty, 2013; Smith et al., 2017), such that our study provides further insights into entrepreneurial intention research by considering the digital transformation context. Although we do not compare online and face-to-face networks, our results suggest a greater importance of online networks than face-to-face networks for entrepreneurial intention. This could be explained by the fact that individuals have many more contacts in online networks than in face-to-face ones (Ellison et al., 2014), and obtain more knowledge and information for promoting innovation (Pérez-González et al., 2017). Furthermore, our results confirm a partially mediating effect of social network size (both online and face-to-face) on the relationship between positive dispositional affectivity and entrepreneurial intention. However, this mediating effect is not important in the case of negative dispositional affectivity. As explained, negative dispositional affectivity influences neither intention nor social network size, such that its mediating effect cannot be significant. Therefore, we go one step further than previous literature’s suggestion regarding the possible indirect impact of affect on entrepreneurship when developing social networks (Baron, 2008; Hayton and Cholakova, 2012), and confirm that only positive affect (and not negative affect) influence entrepreneurship both directly and indirectly (through social networks). This result is in line with Baron (2007), who considers that positive affect is a more important trait for entrepreneurship than negative affect. Anyway, we cannot forget that the direct effect of positive dispositional affectivity is much greater than the indirect effect. This suggests that, in terms of entrepreneurial intention, individuals at first use the interaction of affect and cognition as an internal and even unconscious process. They then consider how this affect is shaped by the social environment, as a more external process, in order to influence cognition. It also suggests that the impact of affect on entrepreneurial intention is partly based on an objective/measurable variable, social network size, and on a more direct path through the influence of affect on individuals’ perceptions and expectations, which may be biased.

### Practical Implications

Beyond its theoretical contribution, our study has practical implications. First, we show that both dispositional affectivities and social networks (face-to-face and online) are important as drivers of entrepreneurial intention and, thus, for the design of specific training programs by institutions that promote entrepreneurial action and disruptive innovations (Fayolle et al., 2006). For example, universities should promote the development of social and psychological abilities among business students, by studying topics related to social psychology. By developing such social skills, students could understand the complementary nature of face-to-face and online networks and the importance of positive dispositional affectivity in order to further these networks and promote entrepreneurial intention and disruptive innovation. Additionally, our finding that positive dispositional affectivity (and not negative dispositional affectivity) has two different paths for influencing entrepreneurial intention, either directly in a subjective way or through social networks in a more objective manner, can be used by different institutions that support entrepreneurship. When individuals apply for funding to start up, these institutions can analyze their dispositional affectivities, and to what extent these individuals use them to develop their entrepreneurial intentions directly or through social networks. This will help choose which individuals are best suited to undertaking entrepreneurial projects that will lead to disruptive innovations.

### Limitations

Our research has several limitations. First, the relationship among dispositional affectivities, networks and cognition is likely to be complex and multidirectional. Despite evidencing certain benefits, our cross-sectional analysis only allows us to study one causal direction of the suggested relations. Although these relations are based on theoretical arguments, future research could evaluate them through longitudinal research. Second, our study focuses on a sample of university students because they have specialized knowledge and competences (Galloway and Brown, 2002), especially in terms of new technologies and Internet (Venkatesh and Morris, 2000), which can favor disruptive innovations. Furthermore, university student samples have the advantage of evaluating individuals who are the same age and who display the same skills, thus endowing the sample with homogeneity. However, precisely because they are all students means that we are unable to know whether the results would also apply to broader samples of individuals. Future research may analyze our model in other samples, particularly of individuals who have already finished their university degrees or vocational education. Eventually, the factors of positive and negative dispositional affectivities are not completely equal to previous scales such as PANAS or PANAS-X. However, second-order modeling allows us to test our hypotheses correctly.

### Future Research

Our research points to several future lines of enquiry. First, we could expand this study by evaluating the resources obtained in social networks, given that the literature suggests that these networks allow individuals to acquire different resources (Jack, 2010). For instance, social networks can provide social support (Renzulli and Aldrich, 2005). Indeed, as previously mentioned, our results suggest a greater importance of online networks than face-to-face networks for entrepreneurial intention. Future studies could try to ascertain if there are specific differences between online and face-to-face social networks in terms of the resources obtained in these two types of social networks that might explain the former’s greater importance. For example, online social networks may offer advantages such as lower uncertainty and higher perceived differentiation (Fischer and Reuber, 2014), which would make it easier for individuals to achieve disruptive innovations than in face-to-face social networks. In addition, although positive affect has several positive consequences, previous research has considered that too much positive affect may also have disadvantages (Baron et al., 2012). Scholars might consider exploring whether social networks developed by high positive affect entrepreneurs really do contribute (or not) to the success (or failure) of a new company in terms of growth or innovations. Furthermore, previous research has proposed that entrepreneurial passion, an intense positive feeling related with entrepreneurship (Cardon et al., 2009), influences entrepreneurial intention (Biraglia and Kadile, 2017). Entrepreneurial passion and dispositional affectivities are likely to work together to also influence social networks. Since entrepreneurial passion is contagious (Cardon et al., 2009), if individuals display entrepreneurial passion in social networks, they could increase the size of these social networks. Future research might integrate dispositional affectivities, entrepreneurial passion, social networks, and entrepreneurial intention in order to obtain more disruptive innovations. Additionally, the field of entrepreneurial intention would benefit from a more dynamic study perspective. For example, the socially situated cognition approach advocates analyzing the interactive psychological processes that link individuals to their environments and vice versa (Smith and Semin, 2006), which is an approach that has previously been used in entrepreneurship research (Cacciotti et al., 2016). Finally, from a broader perspective, existing research has asked for studying how disruptive innovations are transformed to entrepreneurship (Si et al., 2020). Therefore, future research could study if individuals with higher entrepreneurial intentions are better able to take that step from disruptive innovation to entrepreneurship.

## Data Availability Statement

The raw data supporting the conclusions of this article will be made available by the authors, without undue reservation.

## Ethics Statement

Ethical review and approval was not required for the study on human participants in accordance with the local legislation and institutional requirements. The patients/participants provided their written informed consent to participate in this study.

## Author Contributions

All authors contributed to the article and approved the submitted version.

## Conflict of Interest

The authors declare that the research was conducted in the absence of any commercial or financial relationships that could be construed as a potential conflict of interest.

## Footnotes

1 Due to the cross-sectional nature of our study and although there are possible two-way causal relationships between constructs, our theoretical arguments suggest us a direction for causality. In addition, we consider dispositional affectivities as starting point because these dispositions are relatively stable and allow individuals to exhibit a certain kind of response across various situations (Watson and Clark, 1984).

## References

[B1] Ajzen I. (1991). The theory of planned behavior. *Organ. Behav. Hum. Dec. Process.* 50 179–211.

[B2] Ambady N. Gray H. M. (2002). On being sad and mistaken: mood effects on the accuracy of thin-slice judgments. *J. Pers. Soc. Psychol.* 83 947–961. 10.1037/0022-3514.83.4.947 12374446

[B3] Andersson M. A. (2012). Dispositional optimism and the emergence of social network diversity. *Sociol. Q.* 53 92–115. 10.1111/j.1533-8525.2011.01227.x

[B4] Audet J. (2004). A longitudinal study of the entrepreneurial intentions of University Students. *Acad. Entrepreneurship J.* 10 3–16.

[B5] Bae T. J. Qian S. Miao C. Fiet J. O. (2014). The relationship between entrepreneurship education and entrepreneurial intentions: a meta–analytic review. *Entrepreneurship Theory Pract.* 38 217–254. 10.1111/etap.12095

[B6] Bandura A. (1986). *Social Foundations of Thought and Action: A Social Cognitive Theory.* Englewood Cliffs, NJ: Prentice-Hall.

[B7] Baron R. A. (2007). Behavioral and cognitive factors in entrepreneurship: entrepreneurs as the active element in new venture creation. *Strategic Entrepreneurship J.* 1 167–182. 10.1002/sej.12

[B8] Baron R. A. (2008). The role of affect in the entrepreneurial process. *Acad. Manag. Rev.* 33 328–340. 10.5465/amr.2008.31193166

[B9] Baron R. A. Hmieleski K. M. Henry R. A. (2012). Entrepreneurs’ dispositional positive affect: the potential benefits–and potential costs–of being “up”. *J. Bus. Venturing* 27 310–324. 10.1016/j.jbusvent.2011.04.002

[B10] Barsade S. G. (2002). The ripple effect: Emotional contagion and its influence on group behavior. *Administr. Sci. Q.* 47 644–675. 10.2307/3094912

[B11] Baym N. K. (2010). *Personal Connections in the Digital Age.* Cambridge, MA: Polity.

[B12] Berry D. S. Hansen J. S. (1996). Positive affect, negative affect, and social interaction. *J. Pers. Soc. Psychol.* 71:796 10.1037/0022-3514.71.4.796

[B13] Biraglia A. Kadile V. (2017). The role of entrepreneurial passion and creativity in developing entrepreneurial intentions: insights from American homebrewers. *J. Small Bus. Manag.* 55 170–188. 10.1111/jsbm.12242

[B14] Bird B. (1988). Implementing entrepreneurial ideas: the case for intention. *Acad. Manag. Rev.* 13 442–453. 10.2307/258091

[B15] Bless H. Clore G. L. Schwarz N. Golisano V. Rabe C. Wölk M. (1996). Mood and the use of scripts: does a happy mood really lead to mindlessness? *J. Pers. Soc. Psychol.* 71:665. 10.1037/0022-3514.71.4.665 8888596

[B16] Bonesso S. Gerli F. Pizzi C. Cortellazzo L. (2018). Students’ entrepreneurial intentions: the role of prior learning experiences and emotional, social, and cognitive competencies. *J. Small Bus. Manag.* 56 215–242. 10.1111/jsbm.12399

[B17] Bosma H. Stansfeld S. A. Marmot M. G. (1998). Job control, personal characteristics, and heart disease. *J. Occup. Health Psychol.* 3 402–409. 10.1037/1076-8998.3.4.402 9805284

[B18] Boyd D. M. Ellison N. B. (2007). Social network sites: definition, history, and scholarship. *J. Comput. Mediated Commun.* 13 210–230. 10.1111/j.1083-6101.2007.00393.x

[B19] Burt R. S. (2000). The network structure of social capital. *Res. Organ. Behav.* 22 345–423.

[B20] Cacciotti G. Hayton J. C. Mitchell J. R. Giazitzoglu A. (2016). A reconceptualization of fear of failure in entrepreneurship. *J. Bus. Venturing* 31 302–325. 10.1016/j.jbusvent.2016.02.002

[B21] Candi M. Roberts D. L. Marion T. Barczak G. (2018). Social strategy to gain knowledge for innovation. *Br. J. Manag*. 29, 731–749. 10.1111/1467-8551.12280

[B22] Cardon M. S. Wincent J. Singh J. Drnovsek M. (2009). The nature and experience of entrepreneurial passion. *Acad. Manag. Rev.* 34 511–532. 10.5465/amr.2009.40633190

[B23] Chandra Y. Yang S. J. S. (2011). Managing disruptive innovation: entrepreneurial strategies and tournaments for corporate longevity. *J. Gen. Manag.* 37 23–50. 10.1177/030630701103700202

[B24] Chin W. W. Newsted P. R. (1999). Structural equation modeling analysis with small samples using partial least squares. *Stat. Strateg. Small Sample Res*. 1, 307–341.

[B25] Christensen C. M. McDonald R. Altman E. J. Palmer J. (2016). *Disruptive Innovation: Intellectual History and Future Paths.* Cambridge, MA: Harvard Business School, 1–52.

[B26] Corbett A. C. (2007). Learning asymmetries and the discovery of entrepreneurial opportunities. *J. Bus. Venturing* 22 97–118. 10.1016/j.jbusvent.2005.10.001

[B27] Cunningham M. R. (1988). Does happiness mean friendliness? Induced mood and heterosexual self-disclosure. *Pers. Soc. Psychol. Bull.* 14 283–297. 10.1177/0146167288142007 30045476

[B28] Curran J. Blackburn R. A. (2001). Older people and the enterprise society: age and self-employment propensities. *Work Empl. Soc*. 15, 889–902. 10.1177/095001701400438279

[B29] De Carolis D. M. Litzky B. E. Eddleston K. A. (2009). Why networks enhance the progress of new venture creation: the influence of social capital and cognition. *Entrepreneurship Theory Pract.* 33 527–545. 10.1111/j.1540-6520.2009.00302.x

[B30] De Carolis D. M. Saparito P. (2006). Social capital, cognition, and entrepreneurial opportunities: a theoretical framework. *Entrepreneurship Theory Pract.* 30 41–56. 10.1111/j.1540-6520.2006.00109.x

[B31] Delgado-García J. B. De Quevedo-Puente E. Blanco-Mazagatos V. (2015). How affect relates to entrepreneurship: a systematic review of the literature and research agenda. *Int. J. Manag. Rev.* 17 191–211. 10.1111/ijmr.12058

[B32] Direnfeld D. M. Roberts J. E. (2006). Mood congruent memory in dysphoria: the roles of state affect and cognitive style. *Behav. Res. Ther.* 44 1275–1285. 10.1016/j.brat.2005.03.014 16325761

[B33] Donath J. Boyd D. (2004). Public displays of connection. *Technol. J.* 22 71–82. 10.1023/b:bttj.0000047585.06264.cc

[B34] Dubini P. Aldrich H. (1991). Personal and extended networks are central to the entrepreneurial process. *J. Bus. Venturing* 6 305–313. 10.1016/0883-9026(91)90021-5

[B35] Edosomwan S. Prakasan S. K. Kouame D. Watson J. Seymour T. (2011). The history of social media and its impact on business. *J. Appl. Manag. Entrepreneurship* 16 79–91.

[B36] Ellison N. B. Steinfield C. Lampe C. (2011). Connection strategies: social capital implications of Facebook-enabled communication practices. *New Media Soc.* 13 873–892. 10.1177/1461444810385389

[B37] Ellison N. B. Vitak J. Gray R. Lampe C. (2014). Cultivating social resources on social network sites: facebook relationship maintenance behaviors and their role in social capital processes. *J. Comput. Mediated Commun.* 19 855–870. 10.1111/jcc4.12078

[B38] Fayolle A. Gailly B. Lassas-Clerc N. (2006). Assessing the impact of entrepreneurship education programmes: a new methodology. *J. Eur. Ind. Train.* 30 701–720. 10.1108/03090590610715022

[B39] Fayolle A. Liñán F. (2014). The future of research on entrepreneurial intentions. *J. Bus. Res.* 67 663–666. 10.1016/j.jbusres.2013.11.024

[B40] Fischer E. Reuber A. R. (2011). Social interaction via new social media:(How) can interactions on Twitter affect effectual thinking and behavior? *J. Bus. Venturing* 26 1–18. 10.1016/j.jbusvent.2010.09.002

[B41] Fischer E. Reuber A. R. (2014). Online entrepreneurial communication: mitigating uncertainty and increasing differentiation via Twitter. *J. Bus. Venturing* 29 565–583. 10.1016/j.jbusvent.2014.02.004

[B42] Fitzsimmons J. R. Douglas E. J. (2011). Interaction between feasibility and desirability in the formation of entrepreneurial intentions. *J. Bus. Venturing* 26 431–440. 10.1016/j.jbusvent.2010.01.001

[B43] Fodor O. C. Pintea S. (2017). The “emotional side” of entrepreneurship: a meta-analysis of the relation between positive and negative affect and entrepreneurial performance. *Front. Psychol.* 8:310. 10.3389/fpsyg.2017.00310 28348534 PMC5346553

[B44] Foo M. D. (2011). Emotions and entrepreneurial opportunity evaluation. *Entrepreneurship Theory Pract.* 35 375–393. 10.1111/j.1540-6520.2009.00357.x

[B45] Foo M. D. Uy M. A. Murnieks C. (2015). Beyond affective valence: untangling valence and activation influences on opportunity identification. *Entrepreneurship Theory Pract.* 39 407–431. 10.1111/etap.12045

[B46] Forgas J. P. (1995). Mood and judgment: the affect infusion model (AIM). *Psychol. Bull.* 117 39–66. 10.1037/0033-2909.117.1.39 7870863

[B47] Fornell C. Larcker D. F. (1981). Structural equation models with unobservable variables and measurement error: algebra and statistics. *J. Mark. Res.* 18 382–388. 10.2307/3150980

[B48] Frijda N. H. (1986). *The Emotions.* Cambridge, MA: Cambridge University Press.

[B49] Galloway L. Brown W. (2002). Entrepreneurship education at university: a driver in the creation of high growth firms? *Educ. Train.* 44 398–405. 10.1108/00400910210449231

[B50] Gaudreau P. Sanchez X. Blondin J. P. (2006). Positive and negative affective states in a performance-related setting: testing the factorial structure of the PANAS across two samples of French-Canadian participants. *Eur. J. Psychol. Assess.* 22 240–249. 10.1027/1015-5759.22.4.240

[B51] Gedajlovic E. Honig B. Moore C. B. Payne G. T. Wright M. (2013). Social capital and entrepreneurship: a schema and research agenda. *Entrepreneurship Theory Pract.* 37 455–478. 10.1111/etap.12042

[B52] Geers A. L. Reilley S. P. Dember W. N. (1998). Optimism, pessimism, and friendship. *Curr. Psychol.* 17 3–19. 10.1007/s12144-998-1017-4

[B53] Gil de Zúñiga H. Barnidge M. Scherman A. (2017). Social media social capital, offline social capital, and citizenship: exploring asymmetrical social capital effects. *Polit. Commun.* 34 44–68. 10.4324/9780429459955-4

[B54] Global Entrepreneurship Monitor (2018). Available online at: https://www.gemconsortium.org/

[B55] Global University Entrepreneurial Spirit Student’s Survey (2018). Available online at: https://www.guesssurvey.org/publications/publications/international-reports.html

[B56] Greve A. Salaff J. W. (2003). Social networks and entrepreneurship. *Entrepreneurship Theory Pract.* 28 1–22. 10.1111/1540-8520.00029

[B57] Grichnik D. Smeja A. Welpe I. (2010). The importance of being emotional: how do emotions affect entrepreneurial opportunity evaluation and exploitation? *J. Econ. Behav. Organ.* 76 15–29. 10.1016/j.jebo.2010.02.010

[B58] Hair J. F. Jr. Hult G. T. M. Ringle C. Sarstedt M. (2016). *A Primer on Partial Least Squares Structural Equation Modeling (PLS-SEM).* Thousand Oaks, CA: SAGE.

[B59] Harker L. Keltner D. (2001). Expressions of positive emotion in women’s college yearbook pictures and their relationship to personality and life outcomes across adulthood. *J. Pers. Soc. Psychol.* 80:112 10.1037/0022-3514.80.1.112 11195884

[B60] Hayton J. C. Cholakova M. (2012). The role of affect in the creation and intentional pursuit of entrepreneurial ideas. *Entrepreneurship Theory Pract.* 36 41–67. 10.1111/j.1540-6520.2011.00458.x

[B61] Hmieleski K. M. Baron R. A. (2009). Entrepreneurs’ optimism and new venture performance: a social cognitive perspective. *Acad. Manag. J.* 52 473–488. 10.5465/amj.2009.41330755

[B62] Hoang H. Antoncic B. (2003). Network-based research in entrepreneurship: a critical review. *J. Bus. Venturing* 18 165–187.

[B63] Hsu D. K. Simmons S. A. Wieland A. M. (2017). Designing entrepreneurship experiments: a review, typology, and research agenda. *Organ. Res. Methods* 20 379–412. 10.1177/1094428116685613

[B64] Ilouga S. N. Mouloungni A. N. Sahut J. M. (2014). Entrepreneurial intention and career choices: the role of volition. *Small Bus. Econ*. 42 717–728. 10.1007/s11187-013-9524-6

[B65] Isen A. M. Johnson M. M. Mertz E. Robinson G. F. (1985). The influence of positive affect on the unusualness of word associations. *J. Pers. Soc. Psychol.* 48:1413. 10.1037/0022-3514.48.6.1413 4020605

[B66] Isen A. M. Shalker T. E. (1982). Do you “accentuate the positive, eliminate the negative” when you are in a good mood. *Soc. Psychol. Q.* 45 58–63. 10.2307/3033676

[B67] Isen A. M. Shalker T. E. Clark M. Karp L. (1978). Affect, accessibility of material in memory, and behavior: a cognitive loop? *J. Pers. Soc. Psychol.* 36 1–12. 10.1037/0022-3514.36.1.1 621625

[B68] Jack S. L. (2005). The role, use and activation of strong and weak network ties: a qualitative analysis. *J. Manag. Stud*. 42 1233–1259. 10.1111/j.1467-6486.2005.00540.x

[B69] Jack S. L. (2010). Approaches to studying networks: implications and outcomes. *J. Bus. Venturing* 25 120–137. 10.1016/j.jbusvent.2008.10.010

[B70] Johannisson B. (1988). Business formation—a network approach. *Scand. J. Manag.* 4 83–99. 10.1016/0956-5221(88)90002-4

[B71] Jorgensen P. F. (1996). “Affect, persuasion, and communication processes,” in *Handbook of Communication and Emotion*, eds Andersen P. A. Guerrero L. K. (Amsterdam: Elsevier), 403–422. 10.1016/b978-012057770-5/50017-5

[B72] Karimi S. Biemans H. J. Lans T. Chizari M. Mulder M. (2016). The impact of entrepreneurship education: a study of Iranian students’ entrepreneurial intentions and opportunity identification. *J. Small Bus. Manag.* 54 187–209. 10.1111/jsbm.12137

[B73] Keltner D. (1996). Evidence for the distinctness of embarrassment, shame, and guilt: a study of recalled antecedents and facial expressions of emotion. *Cogn. Emot.* 10 155–172. 10.1080/026999396380312

[B74] Kim P. Aldrich H. (2005). *Social Capital and Entrepreneurship*. Boston: Now Publishers Inc.

[B75] Kleinbaum D. Kupper L. Nizam A. Rosenberg E. (2013). *Applied Regression Analysis and Other Multivariable Methods.* Boston: Thomson Higher Education.

[B76] Kolvereid L. (1996). Prediction of employment status choice intentions. *Entrepreneurship Theory Pract.* 21 47–58. 10.1177/104225879602100104

[B77] Krueger N. (1993). The impact of prior entrepreneurial exposure on perceptions of new venture feasibility and desirability. *Entrepreneurship Theory Pract.* 18 5–21. 10.1177/104225879301800101

[B78] Krueger N. F. Jr. Reilly M. D. Carsrud A. L. (2000). Competing models of entrepreneurial intentions. *J. Bus. Venturing* 15 411–432. 10.1016/s0883-9026(98)00033-0

[B79] Kunda Z. (1999). *Social Cognition: Making Sense of People.* Cambridge, MA: MIT press.

[B80] Lazarus R. S. (1982). Thoughts on the relations between emotion and cognition. *Am. Psychol.* 37:1019 10.1037/0003-066x.37.9.1019

[B81] Lerner J. S. Keltner D. (2000). Beyond valence: toward a model of emotion-specific influences on judgement and choice. *Cogn. Emot.* 14 473–493. 10.1080/026999300402763

[B82] Leyden D. P. Link A. N. Siegel D. S. (2014). A theoretical analysis of the role of social networks in entrepreneurship. *Res. Policy* 43 1157–1163. 10.1016/j.respol.2014.04.010

[B83] Liñán F. Chen Y. W. (2009). Development and cross–cultural application of a specific instrument to measure entrepreneurial intentions. *Entrepreneurship Theory Pract.* 33 593–617. 10.1111/j.1540-6520.2009.00318.x

[B84] Liñán F. Urbano D. Guerrero M. (2011). Regional variations in entrepreneurial cognitions: start-up intentions of university students in Spain. *Entrepreneurship Regional Dev.* 23 187–215. 10.1080/08985620903233929

[B85] Lucas R. E. Diener E. (2003). The happy worker: hypotheses about the role of positive affect in worker productivity. *Pers. Work* 9 30–59.

[B86] Lyubomirsky S. King L. Diener E. (2005). The benefits of frequent positive affect: does happiness lead to success? *Psychol. Bull.* 131:803. 10.1037/0033-2909.131.6.803 16351326

[B87] Mathews C. H. Moser S. B. (1995). Family background and gender: implications for interest in small firm ownership. *Entrepreneurship Regional Dev.* 7 365–378. 10.1080/08985629500000023

[B88] Mor N. Winquist J. (2002). Self-focused attention and negative affect: a meta-analysis. *Psychol. Bull.* 128:638. 10.1037/0033-2909.128.4.638 12081086

[B89] Nikolaev B. Shir N. Wiklund J. (2019). Dispositional positive and negative affect and self-employment transitions: the mediating role of job satisfaction. *Entrepreneurship Theory Pract.* 44:1042258718818357.

[B90] Papa A. Santoro G. Tirabeni L. Monge F. (2018). Social media as tool for facilitating knowledge creation and innovation in small and medium enterprises. *Baltic J. Manag*. 13, 329–344. 10.1108/BJM-04-2017-0125

[B91] Pérez-González D. Trigueros-Preciado S. Popa S. (2017). Social media technologies’ use for the competitive information and knowledge sharing, and its effects on industrial SMEs’ innovation. *Inform. Syst. Manag.* 34 291–301. 10.1080/10580530.2017.1330007

[B92] Podsakoff P. M. MacKenzie S. B. Lee J. Podsakoff N. P. (2003). Common method biases in behavioral research: a critical review of the literature and recommended remedies. *J. Appl. Psychol.* 88:879. 10.1037/0021-9010.88.5.879 14516251

[B93] Podsakoff P. M. Organ D. W. (1986). Self-reports in organizational research: problems and prospects. *J. Manag.* 12 531–544. 10.1177/014920638601200408

[B94] Räikkönen K. Matthews K. A. Flory J. D. Owens J. F. Gump B. B. (1999). Effects of optimism, pessimism, and trait anxiety on ambulatory blood pressure and mood during everyday life. *J. Pers. Soc. Psychol.* 76:104. 10.1037/0022-3514.76.1.104 9972556

[B95] Rainie L. Wellman B. (2012). *Networked: The New Social Operating System.* Cambridge, MA: MIT Press.

[B96] Renzulli L. A. Aldrich H. (2005). Who can you turn to? Tie activation within core business discussion networks. *Soc. Forces* 84 323–341. 10.1353/sof.2005.0122

[B97] Requena F. (1995). Friendship and subjective well-being in Spain: a cross-national comparison with the United States. *Soc. Indic. Res.* 35 271–288. 10.1007/bf01079161

[B98] Reuber A. R. Fischer E. (2011). International entrepreneurship in Internet-enabled markets. *J. Bus. Venturing* 26 660–679. 10.1016/j.jbusvent.2011.05.002

[B99] Roldán J. L. Leal A. (2003). “A validation test of an adaptation of the delone and McLean’s model in the spanish EIS field,” in *Critical Reflections on Information Systems: A Systemic Approach*, ed. Cano J. J. (Pennsylvania: IGI Global), 66–84. 10.4018/978-1-59140-040-0.ch004

[B100] Rusting C. L. (1998). Personality, mood, and cognitive processing of emotional information: three conceptual frameworks. *Psychol. Bull.* 124:165. 10.1037/0033-2909.124.2.165 9747185

[B101] Sandín B. Chorot P. Lostao L. Joiner T. E. Santed M. A. Valiente R. M. (1999). Escalas PANAS de afecto positivo y negativo: validación factorial y convergencia transcultural. *Psicothema* 11 37–51.

[B102] Schumpeter J. A. (1934). The Theory of Economic Development. Cambridge: HarvardUniversity Press.

[B103] Scuotto V. Del Giudice M. Della Peruta M. R. Tarba S. (2017). The performance implications of leveraging internal innovation through social media networks: an empirical verification of the smart fashion industry. *Technol. Forecast. Soc. Change* 120, 184–194. 10.1016/j.techfore.2017.03.021

[B104] Shapero A. Sokol L. (1982). “Social dimensions of entrepreneurship,” in *The Encyclopedia of Entrepreneurship*, eds Kent C. Sexton D. Vesper K. (Englewood Cliffs: Prentice-Hall), 72–90.

[B105] Shepherd D. A. (2003). Learning from business failure: propositions of grief recovery for the self-employed. *Acad. Manag. Rev.* 28 318–328. 10.2307/30040715

[B106] Si S. Zahra S. A. Wu X. Jeng D. J. F. (2020). Disruptive innovation and entrepreneurship in emerging economics. *J. Eng. Technol. Manag.* 58:101601 10.1016/j.jengtecman.2020.101601

[B107] Sigfusson T. Chetty S. (2013). Building international entrepreneurial virtual networks in cyberspace. *J. World Bus.* 48 260–270. 10.1016/j.jwb.2012.07.011

[B108] Simon M. Houghton S. M. Aquino K. (2000). Cognitive biases, risk perception, and venture formation: how individuals decide to start companies. *J. Bus. Ventur*. 15, 113–134.

[B109] Smith C. Smith J. B. Shaw E. (2017). Embracing digital networks: entrepreneurs’ social capital online. *J. Bus. Venturing* 32 18–34. 10.1016/j.jbusvent.2016.10.003

[B110] Smith E. R. Semin G. R. (2006). “Socially situated cognition as a bridge,” in *Bridging Social Psychology: Benefits of Transdisciplinary Approaches*, ed. van Lange P. (Milton Park: Taylor & Francis Group), 145 ed. van Lange P. (Milton Park: Taylor & Francis Group), 145–150.

[B111] Srivastava A. Bartol K. M. Locke E. A. (2006). Empowering leadership in management teams: effects on knowledge sharing, efficacy, and performance. *Acad. Manag. J.* 49 1239–1251. 10.5465/amj.2006.23478718

[B112] Staw B. M. Sutton R. I. Pelled L. H. (1994). Employee positive emotion and favorable outcomes at the workplace. *Organ. Sci.* 5 51–71. 10.1287/orsc.5.1.51 19642375

[B113] Steinfield C. Ellison N. B. Lampe C. (2008). Social capital, self-esteem, and use of online social network sites: a longitudinal analysis. *J. Appl. Dev. Psychol.* 29 434–445. 10.1016/j.appdev.2008.07.002

[B114] Subrahmanyam K. Reich S. M. Waechter N. Espinoza G. (2008). Online and offline social networks: use of social networking sites by emerging adults. *J. Appl. Dev. Psychol*. 29, 420–433. 10.1016/j.appdev.2008.07.003

[B115] Venkatesh V. Morris M. G. (2000). Why don’t men ever stop to ask for directions? Gender, social influence, and their role in technology acceptance and usage behavior. *MIS Q.* 24 115–139. 10.2307/3250981

[B116] Watson D. Clark L. A. (1984). Negative affectivity: the disposition to experience aversive emotional states. *Psychol. Bull.* 96 465–490. 10.1037/0033-2909.96.3.465 6393179

[B117] Watson D. Clark L. A. (1999). *The PANAS-X: Manual for the Positive and Negative Affect Schedule-Expanded Form.* Ames: The University of Iowa.

[B118] Watson D. Clark L. A. McIntyre C. W. Hamaker S. (1992). Affect, personality, and social activity. *J. Pers. Soc. Psychol.* 63:1011. 10.1037/0022-3514.63.6.1011 1460554

[B119] Watson D. Clark L. A. Tellegen A. (1988). Development and validation of brief measures of positive and negative affect: the PANAS scales. *J. Pers. Soc. Psychol.* 54:1063. 10.1037/0022-3514.54.6.1063 3397865

[B120] Wellman B. Haase A. Q. Witte J. Hampton K. (2001). Does the Internet increase, decrease, or supplement social capital? Social networks, participation, and community commitment. *Am. Behav. Sci.* 45 436–455. 10.1177/00027640121957286

[B121] Wittmann C. M. Hunt S. D. Arnett D. B. (2009). Explaining alliance success: competences, resources, relational factors, and resource-advantage theory. *Ind. Mark. Manag.* 38 743–756. 10.1016/j.indmarman.2008.02.007

[B122] Wood R. Bandura A. (1989). Social cognitive theory of organizational management. *Acad. Manag. Rev.* 14 361–384. 10.2307/258173

[B123] Wright W. F. Bower G. H. (1992). Mood effects on subjective probability assessment. *Organ. Behav. Hum. Dec. Process.* 52 276–291. 10.1016/0749-5978(92)90039-a

[B124] Zajonc R. B. (1980). Feeling and thinking: preferences need no inferences. *Am. Psychol.* 35:151 10.1037/0003-066x.35.2.151

[B125] Zelenski J. M. Larsen R. J. (2002). Predicting the future: how affect-related personality traits influence likelihood judgments of future events. *Pers. Soc. Psychol. Bull.* 28 1000–1010. 10.1177/014616720202800712

